# An Integrated System for Precise Genome Modification in *Escherichia coli*

**DOI:** 10.1371/journal.pone.0136963

**Published:** 2015-09-02

**Authors:** Huseyin Tas, Cac T. Nguyen, Ravish Patel, Neil H. Kim, Thomas E. Kuhlman

**Affiliations:** 1 Center for the Physics of Living Cells, University of Illinois at Urbana-Champaign, Urbana, Illinois, United States of America; 2 Center for Biophysics and Computational Biology, University of Illinois at Urbana-Champaign, Urbana, Illinois, United States of America; 3 Department of Physics, University of Illinois at Urbana-Champaign, Urbana, Illinois, United States of America; 4 Department of Molecular and Cellular Biology, University of Illinois at Urbana-Champaign, Urbana, Illinois, United States of America; Baylor College of Medicine, UNITED STATES

## Abstract

We describe an optimized system for the easy, effective, and precise modification of the *Escherichia coli* genome. Genome changes are introduced first through the integration of a 1.3 kbp Landing Pad consisting of a gene conferring resistance to tetracycline (*tetA*) or the ability to metabolize the sugar galactose (*galK*). The Landing Pad is then excised as a result of double-strand breaks by the homing endonuclease I-SceI, and replaced with DNA fragments bearing the desired change via λ-Red mediated homologous recombination. Repair of the double strand breaks and counterselection against the Landing Pad (using NiCl_2_ for *tetA* or 2-deoxy-galactose for *galK*) allows the isolation of modified bacteria without the use of additional antibiotic selection. We demonstrate the power of this method to make a variety of genome modifications: the exact integration, without any extraneous sequence, of the *lac* operon (~6.5 kbp) to any desired location in the genome and without the integration of antibiotic markers; the scarless deletion of ribosomal *rrn* operons (~6 kbp) through either intrachromosomal or oligonucleotide recombination; and the *in situ* fusion of native genes to fluorescent reporter genes without additional perturbation.

## Introduction

The ability to introduce genes to an organism or alter their coding sequences is one of the fundamental tools of modern biology. While in many cases genes can be easily manipulated and expressed from extrachromosomal plasmids, it is frequently necessary or desirable to edit the organism’s chromosome directly; for example, in the generation of knockout mutants where the coding sequence of a gene is disrupted or eliminated from the organism entirely. As a consequence, a wide variety of tools for the molecular editing of bacterial chromosomes has been developed to generate various types of genomic modifications. These tools employ, for example, homologous recombination (e.g., recombineering, [1], KIKO [2], FRUIT [3], and PLRS [4]), the cleavage or excision of a target genomic sequence through the nuclease activity of homing endonucleases (e.g., gene gorging [5], MAGIC [6], ALFIRE [7], and earlier versions of Landing Pad technology [8, 9]), phage-derived integrases (CRIM, [10]; clonetegration [11]; ΦC31 [12–15]), and CRISPR-Cas9 based systems [16–20]. Additional tools for genome editing in eukaryotes include engineered zinc-finger nucleases (ZFNs, [20, 21]) and transcription activator-like effector nucleases (TALENs, [20, 22, 23]).

In many cases, the system used is dictated by the type of genomic modification desired. Recombineering is well suited for the integration of small DNA fragments into specific genomic locations, making it ideal for introducing point mutations, performing deletions, or integrating short coding sequences. Conversely, phage-based systems are applicable for the integration of large constructs at preexisting phage attachment sites in the genome; however, they are generally inapplicable to other tasks, such as targeting insertions to specific genomic loci or the generation of knockout mutants. Nuclease-assisted recombineering, such as CRISPR-Cas9 systems and Landing Pad technology, combine the benefits of both recombineering and phage-based editing methods: they are able to easily introduce or delete large coding sequences at any desired genomic location and in any orientation. They differ only in how the nuclease activity is targeted to the genomic location to be edited. CRISPR-Cas9 systems require the *in vivo* expression of a guide RNA complementary to the targeted location. The Landing Pad system, on the other hand, is guided by the recombineering of a short “landing pad” including unique recognition sites for the homing endonuclease I-SceI at the desired location.

**Fig 1:**
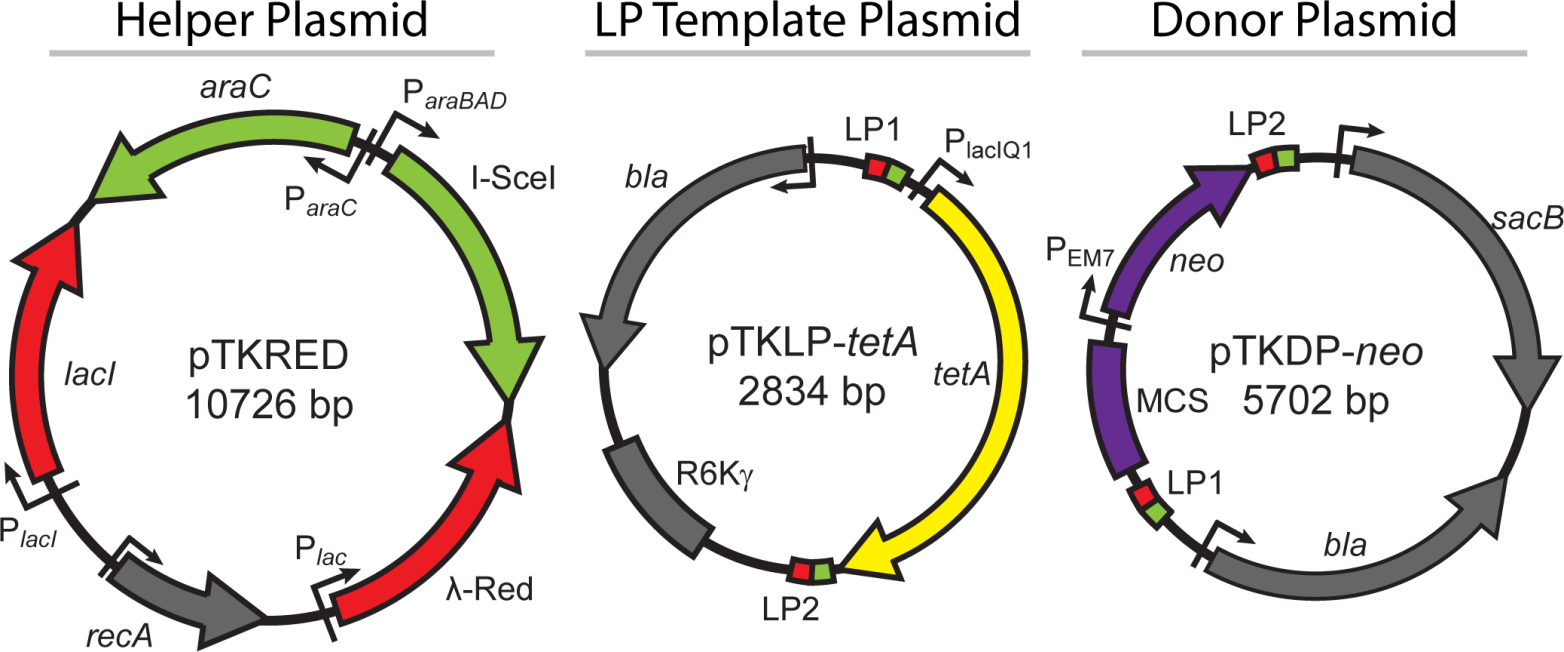
Plasmids of the Landing Pad System. The helper plasmid, pTKRED, expresses I-SceI (inducible with L-arabinose) and the λ-Red enzymes (inducible with IPTG), and has a temperature sensitive pSC101 origin of replication that can be cured by growth at 42°C. The LP template plasmid, pTKLP, serves as a PCR template for amplification of either the *tetA* or *galK* landing pad, and carries a R6Kγ *pir* ^+^-dependent origin of replication. The donor plasmid, pTKDP, serves as a fragment donor for the integration of large constructs that have been cloned into the purple region of the plasmid, guided by recombination with landing pad LP1 and LP2 sequences, or custom homology regions as described in this report. The sequence sizes given are for pTKLP-*tetA* and pTKDP-*neo*; *tetA* is exactly replaced with *galK* in pTKLP-*galK*, and *neo* is exactly replaced with various antibiotic resistance genes for alternate versions of pTKDP. Small green boxes are I-SceI restriction sites; Landing Pad Regions 1 and 2 are small red boxes labeled LP1 and LP2 respectively.

Here, we describe an optimized Landing Pad system [8, 9] of three engineered plasmids (Fig 1) that allows for the precise modification of the *E*. *coli* genome in a wide variety of ways. A helper plasmid, pTKRED [8], allows the inducible expression of the recombinogenic λ-Red enzymes [1, 24–29], the homing endonuclease I-SceI [5, 9, 30], and RecA. The plasmid pTKLP serves as a PCR template for the amplification of a 1.3 kbp “landing pad”, which consists of a gene conferring either resistance to tetracycline (*tetA*) or the ability to metabolize the sugar galactose (*galK*, [1, 5]) flanked by I-SceI recognition sites and standardized sites for priming and homologous recombination (LP1 and LP2 in Fig 1). Because of its small size, the landing pad is easily recombineered into the desired location in the genome [8] by λ-Red mediated homologous recombination, and serves as a subsequent target for cleavage by I-SceI and recombination by λ-Red. Successful integration of the landing pad can be positively selected for through growth on medium containing tetracycline (for *tetA* landing pad) or galactose as the sole carbon source (for *galK* landing pad). Finally, the ultimately desired modification can be accomplished through excision of the landing pad and/or replacement with a DNA fragment carrying the modification; if this requires the integration of a large fragment (> ~2.5 kbp), the donor plasmid pTKDP, which harbors the integration fragment, is co-transformed into the cells and digested *in vivo* by I-SceI. In either case, homologous recombination results in repair or replacement of the excised landing pad with the desired integration fragment or modified sequence.

The efficiency of selection for successfully modified cells is enhanced through negative selection against retention of the landing pad. In the case of *tetA*, the bacteria are inoculated into medium containing NiCl_2_ after the integration step; NiCl_2_ is selectively lethal to *E*. *coli* expressing *tetA* [31], and hence those cells which are unsuccessfully modified and retain the *tetA* landing pad are eliminated from the population. Alternatively, the *galK* landing pad can be selected against by growth of the bacteria in the presence of 2-deoxy-galactose (DOG, [1, 5]). The enzyme product of *galK*, galactokinase, phosphorylates DOG into the non-metabolizable product 2-deoxy-galactose-1-phosphate, which builds up to lethal levels in those cells retaining the landing pad. In particular, the highly efficacious selection and counterselection for *galK* using galactose and DOG [1, 5], combined with the lethality of chromosomal double breaks caused by I-SceI and subsequent rescue by the repair of the break by successful modification [8, 9], allows the precise editing of the genome and integration of large constructs without the accompanying integration of any antibiotic resistance genes into the host genome at any point. Such an antibiotic-free approach can be advantageous when, for example, working with potentially pathogenic strains or species.

Furthermore, the plasmids pTKLP and pTKDP incorporate selectable countermeasures against undesired transformation or retention of the plasmids, eliminating requirements for tedious and time consuming screening [8]. pTKLP contains the R6Kγ origin of replication, and can therefore only be stably transformed and maintained in cells expressing the Pi protein required for R6Kγ replication [10]. As wildtype K-12 MG1655 and many other commonly used *E*. *coli* strains do not express Pi, this ensures that cells positively selected for *tetA* or *galK* expression are the result of successful incorporation of the landing pad rather than transformation of residual pTKLP used as PCR template. For pTKDP, the unintegrated plasmid backbone constitutively expresses levansucrase encoded by the gene *sacB* [32–35], which catalyzes the hydrolysis of sucrose into non-metabolizable levans. Therefore, when sucrose is added to the medium during the counterselection step, those cells which are not cured of pTKDP by I-SceI are lysed as a result of the buildup of levans within the periplasmic space [35].

We demonstrate the power of this single method to perform a variety of genome modifications that previously required the application of a variety of distinct tools and techniques. Specifically, we perform: [1] the integration of the entire *lac* operon (~6.5 kbp) into any desired location of the genome without the introduction of any extraneous sequence or the integration of any antibiotic resistance genes into the genome; [2] the direct, *in situ* fusion of native chromosomal genes to fluorescent reporter genes without any additional perturbation; and [3] the scarless deletion of ribosomal *rrn* operons (~6 kbp) through either intrachromosomal homologous recombination or recombination with oligonucleotides.

## Materials and Methods

### Bacterial strains

Strains used were wildtype *E*. *coli* K-12 MG1655 (Coli Genetic Stock Center). For exact integrations of the entire *lac* operon, an MG1655 strain in which the native *lac* operon has been deleted was used ([8], henceforth referred to as MG1655 Δ*lac*; deletion including genomic locations 361249–367510). For antibiotic-free integrations with *galK* as a selection marker, an MG1655 Δ*lac* strain where the native *galK* sequence was deleted using the method of Datsenko and Wanner [25] was used (referred to throughout as MG1655 Δ*lac* Δ*galK*; deletion including genomic locations 788831–789979). We have additionally generated an MG1655 Δ*galK* strain for general use. Annotated sequences for pTKRED, pTKLP-*tetA*, pTKLP-*galK*, pTKDP-*neo*, pTKDP-*cat*, pTKDP-*hph*, and pTKDP-*dhfr* are available as Genbank accession numbers GU327533, KR071151, KR071150, KR071149, KR071146, KR071148, and KR071147 respectively.

### Preparation of competent cells and transformation for recombineering

Cells competent for transformation by electroporation were prepared by inoculating an overnight culture into 30 ml of Super Optimal Broth (SOB medium) in a baffled 125 ml Erlenmeyer flask with appropriate antibiotics; if the cells were to be used for recombineering with pTKRED, 2 mM IPTG was also added to the medium at the time of inoculation. These cultures were grown at 30°C in a New Brunswick C76 shaking water bath until OD_600_ ~0.6, at which point the cultures were placed on ice. The cells were made electrocompetent by centrifugation (5 min at 5,000 rpm) and washing with sterile ice-cold 10% v/v glycerol three times. 100 μl of competent cells were then mixed with ~100 ng of DNA in a 5 ml polystyrene round bottom tube (Falcon) on ice. This mixture of DNA and competent cells was transferred to a 0.1 cm gap electroporation cuvette (USA Scientific) and shocked at 2.0 kV, 25 uF, 200 Ω in a Bio-Rad Micropulser electroporation apparatus. 1 ml of SOB medium was immediately added and transferred back to the 5 ml Falcon tube; the resulting culture was allowed to recover for four hours in a 30°C shaking water bath. At this point, 500 μl of culture was spun down in a table top microcentrifuge (5 min at 10,000 rpm), the supernatant dumped, the cells resuspended in the residual medium and spread on Lysogeny Broth (LB) plates containing the appropriate antibiotic, and then placed into a 30°C air incubator overnight. The remainder of the culture was allowed to recover overnight at room temp on the benchtop. The next day, if no colonies had grown on the plates, the remainder of the culture was spun down and plated in a similar fashion.

### Construction of the donor plasmid pTKDP

To construct improved versions of the pTKIP plasmid, the donor plasmid in the previously reported integration scheme [8, 9], the *sacB* gene, conferring sensitivity to the sugar sucrose [32–35], and its promoter were amplified from plasmid pKO3 [36] by PCR using primers containing NdeI restriction sites. The PCR product was PCR purified (QIAquick) and digested with NdeI. The plasmid pTKIP-*neo* was also digested with NdeI, dephosphorylated with Antarctic phosphatase (New England Biosciences), and gel purified (QIAquick). The plasmid was ligated together with the *sacB* fragment, forming the plasmid pTKDP-*neo*.

Versions of pTKDP with alternate antibiotic resistances (pTKDP-*cat* chloramphenicol resistant; pTKDP-*hph* hygromycin B resistant; pTKDP-*dhfr* trimethoprim resistant) were constructed through recombineering [1, 25]. First, the alternate antibiotic resistance genes were amplified from the corresponding pTKIP plasmid using primers including 50 bp of the sequence of pTKDP flanking either side of the gene. The resulting PCR product was checked for the correct size via agarose gel electrophoresis and PCR purified. Next, pTKDP was transformed into the recombineering strain SW102 [1], and the resulting strain was transformed with the purified PCR products and plated onto LB agar containing the desired new antibiotic. The new pTKDP plasmids from colonies growing on these plates were purified (QIAprep) and verified by sequencing (ACGT Inc).

### Construction of the landing pad template plasmids pTKLP-*tetA*/*galK*

To construct plasmid pTKLP-*tetA*, the *tetA* landing pad cassette was purified from plasmid pTKS/CS [8] by digestion with I-SceI (NEB) and subsequent gel purification. A plasmid backbone containing the R6Kγ replication origin was amplified from pKD3 (2) using primers including I-SceI restriction sites and the landing pad sequences LP1 and LP2. This backbone was PCR purified, digested with I-SceI, dephosphorylated with Antarctic phosphatase, and gel purified. The backbone and landing pad cassette were ligated together and transformed into strain BW23474 [10], which constitutively expresses the Pi protein required for R6Kγ replication maintenance. Plasmid pTKLP-*galK* was created in a similar fashion. *galK* was amplified from wildtype MG1655 by colony PCR using primers containing I-SceI restriction sites and the strong constitutive promoter P_lacIQ1_ [37]. This cassette was PCR purified and digested with I-SceI and ligated into the same R6Kγ plasmid backbone used for pTKLP-*tetA*.

### Amplification, preparation, and integration of Landing Pad

Linear Landing Pad DNA fragment (LP) was amplified from the plasmid pTKLP by PCR using Phusion Flash High-Fidelity PCR Master Mix (Life Technologies) and locus and application specific primers; a table of all oligonucleotides used for recombineering in this study is given as S1 Table. Both pTKLP-*tetA* and pTKLP-*galK* contain standardized 25 bp priming sequences [Landing Pad Region 1 (LP1): 5’ TACGGCCCCAAGGTCCAAACGGTGA 3’; Landing Pad Region 2 (LP2): 5’ GATGGCGCCTCATCCCTGAAGCCAA 3’] that can also be used as subsequent targets for homologous recombination for general chromosomal integration of constructs [8, 9]. Optimum amplification conditions were decided for each primer set by thermal gradient PCR followed by agarose gel electrophoresis. Samples were PCR purified and digested with DpnI (New England Biolabs) for at least four hours at 37°C to eliminate template plasmid contamination, followed by PCR purification.

To integrate the landing pad, cells were first prepared by transformation with the helper plasmid pTKRED [8]. These cells were then made competent and transformed with the purified landing pad as described above (see *Preparing competent cells and transformation)*. When plating, cells recombineered with the *tetA* landing pad were spread on LB plates with 10 μg/ml tetracycline and 100 μg/ml spectinomycin; for Δ*galK* cells incorporating the *galK* landing pad, cells were plated on M63 minimal medium plates with 0.2% w/v galactose as carbon source and 100 μg/ml spectinomycin [1]. These plates were incubated at 30°C overnight, at which point the remainder of the transformed culture was plated in a similar fashion. Plates were allowed to grow until colonies were visible, requiring ~15 hours for *tetA* integrants and ~48 hours for *galK* integrants on minimal medium plates. Integrants were verified by colony PCR, gel electrophoresis, and sequencing.

### Counterselection against tetA with NiCl_2_ and determination of growth rate

Growth rate experiments for wildtype MG1655 and MG1655 strains with the *tetA* landing pad incorporated at the *rrnB* ribosomal operon (*rrnB*::*tetA*) were performed in a 24 well Corning Costar microplate. To begin, strains were grown overnight in 5 ml of Rich Defined Medium (RDM, Teknova) + 0.5% v/v glycerol at 37°C in separate tubes with appropriate antibiotics. Then, two separate tubes for each strain containing 50 ml of RDM + 0.5% v/v glycerol were prepared by inoculation with the overnight cultures such that the calculated initial OD was 0.002. Wells of the plate were filled with 2 mL of these cultures, and appropriate dilutions of 1 M NiCl_2_ solution were added to the wells to cover concentrations of 0–10 mM NiCl_2_ in 1 mM increments. After preparation, the plate was placed into a Tecan Infinite 200 plate reader (Tecan Ltd) at 37°C with shaking, and OD values at 600 nm were recorded every 15 minutes for 48 hours.

**Fig 2:**
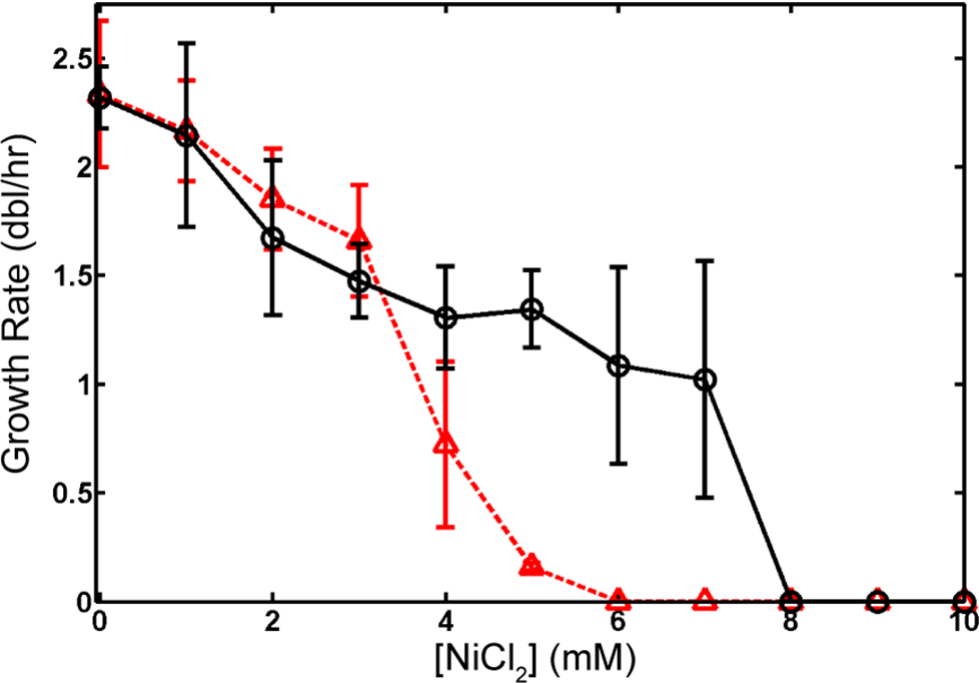
*tetA* counterselection with NiCl_2_. Wildtype MG1655 (black circles, solid line) and MG1655 *rrnB*::*tetA* (red triangles, dashed line) were grown in RDM + 0.5% v/v glycerol with concentrations of NiCl_2_ from 0–10 mM, and the effect of NiCl_2_ quantified by measuring the growth rate (doublings per hour) in exponential growth. Points are the average of three measurements, and error bars are the SD. The growth rate for both strains at high concentrations of NiCl_2_ where no observable growth was detected is set to zero. Because of the large differential in growth rate, counterselection against the *tetA* landing pad can be effectively performed at 5–7 mM NiCl_2_ in K-12 MG1655.

To calculate the doubling time of each well, the data collected from the plate reader were analyzed using MATLAB (MathWorks). Each growth curve was blanked using the average OD_600_ reading collected from two uninoculated control wells. Regions of exponential growth for each curve were identified by visual inspection of plots of Log_2_(OD_600_) vs time and fit by linear regression to extract growth rates for each NiCl_2_ concentration. At high concentrations of NiCl_2_ (> ~8 mM) neither the wildtype nor *tetA* landing pad strain was able to grow and the growth curves were indistinguishable from the background; the growth rates of these cultures were set to zero in Fig 2.

### Construction of fragments for exact integration

To test the efficiency of the exact integration of large constructs without any extraneous sequence or antibiotic markers [9], pTKDP-*neo* was first digested with I-SceI and the products dephosphorylated with Antarctic phosphatase. The plasmid backbone, containing the origin of replication, *bla* ampicillin resistance gene, and the *sacB* counterselection marker, was gel purified from this reaction by gel purification (QIAquick).

The entire *lac* operon, including the promoter of *lacI* and terminators of *lacA*, was amplified from wildtype MG1655 by colony PCR using primers containing I-SceI restriction sites and 50 bp of homology to the targeted genomic location [either the *atpI*, *nth*, or *ygcE* locus [8, 9, 38]]. The PCR reactions were purified, digested with I-SceI, and gel purified. These fragments were then ligated together with the purified pTKDP backbone to form plasmids pTKDP-*atpI*-*lacIZYA*, pTKDP-*nth*-*lacIZYA*, and pTKDP-*ygcE*-*lacIZYA*.

### Exact, antibiotic-free integration

Electrocompetent MG1655 Δ*lac* Δ*galK* carrying the helper plasmid pTKRED and induced to express λ-Red enzymes were prepared and transformed with ~100 ng of purified *galK* landing pad targeted towards either the *atpI*, *nth*, or *ygcE* locus. Successful integrants were obtained by selection on M63 minimal medium plates with 0.2% w/v galactose as the sole carbon source [1] and verified by colony PCR. The resulting strains were transformed with the appropriate pTKDP-*xxx*-*lacIZYA* plasmid and spread on LB + 100 μg/ml ampicillin plates, where *xxx* can be any of the three genomic loci tested (*atpI*, *nth*, or *ygcE*). Several colonies were picked and used to inoculate a 20 mm glass tube containing 5 ml of RDM + 0.5% v/v glycerol medium with 100 μg/ml spectinomycin, 2 mM IPTG to induce expression of λ-Red enzymes, and 0.4% w/v L-arabinose to induce expression of I-SceI. These tubes were allowed to grow in a shaking 30°C water bath until saturation. At this point, a small sample was taken, diluted 10^5^ fold, and plated on LB plates with 2 mM IPTG and 20 μg/ml X-gal and allowed to grow overnight at 37°C. The number of correct integrants was quantified as the number of blue versus white colonies.

**Fig 3:**
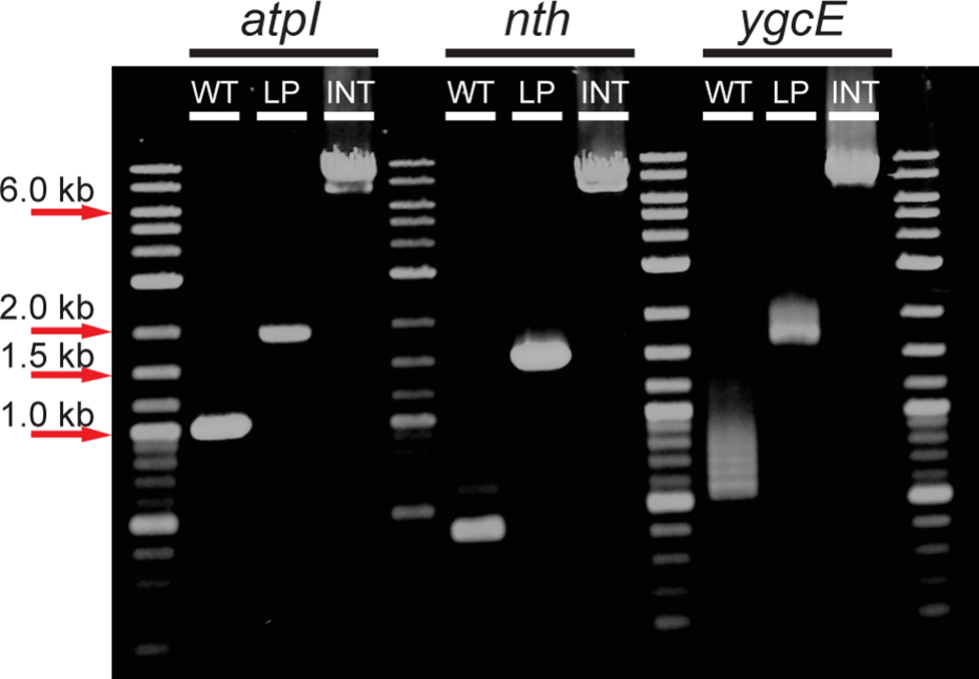
Exact, antibiotic-free integration. Agarose gel electrophoresis of colony PCR products using primers that bind to regions flanking each locus (*atpI*, *nth*, and *ygcE*). The first lanes of each show PCR products from wildtype cells (WT). The second lanes of each show the PCR product after the landing pad is inserted (LP). The last lanes of each show PCR products after integration of the *lac* operon at each locus (INT).

At the same time, 100 μl of the each saturated tube was used to inoculate another glass tube containing 5 ml of RDM + 0.5% glycerol with 0.2% w/v DOG for *galK* counterselection and 5% w/v sucrose for *sacB* counterselection. These tubes were allowed to grow at 37°C until saturated, usually taking 1–2 days. After saturation, a small sample was taken, diluted 10^5^ fold, and plated on LB plates with 2 mM IPTG and 20 μg/ml X-gal and allowed to grow overnight at 37°C. The number of correct integrants was quantified as the number of blue versus white colonies. Several colonies for each integration location were picked and verified by colony PCR (Fig 3).

We also performed this same procedure using the *tetA* landing pad and counterselection with NiCl_2_ to gather statistics on the efficiency of integration (Table 1).

**Table 1:** Exact Integration Statistics.

	Without Counterselection				With Counterselection			
	*tetA* (Blue/White)	% Correct	*galK* (Blue/White)	% Correct	*tetA* (Blue/White)	% Correct	*galK* (Blue/White)	% Correct
*atpI*	888 / 25	97.3	54 / 829	6.1	1148 / 15	98.7	720 / 6	99.2
*nth*	1104 / 64	94.5	49 / 248	16.5	1375 / 35	97.5	601 / 9	98.5
*ygcE*	194 / 559	25.8	51 / 353	12.6	1052 / 306	77.5	232 / 200	53.7

Colony counts resulting from exact integration of the *lac* operon at each of three loci (*atpI*, *nth*, and *ygcE*) using either the *tetA* or *galK* landing pad. Counts are given both before and after counterselection against landing pad retention using 5% w/v sucrose and either 6 mM NiCl_2_ (for *tetA* landing pad) or 0.2% w/v DOG (for *galK* landing pad). The percent correct integrants was calculated as the percentage of blue colonies after growth on 2 mM IPTG and 20 μg/ml X-gal.

### In situ gene fusion

**Fig 4:**
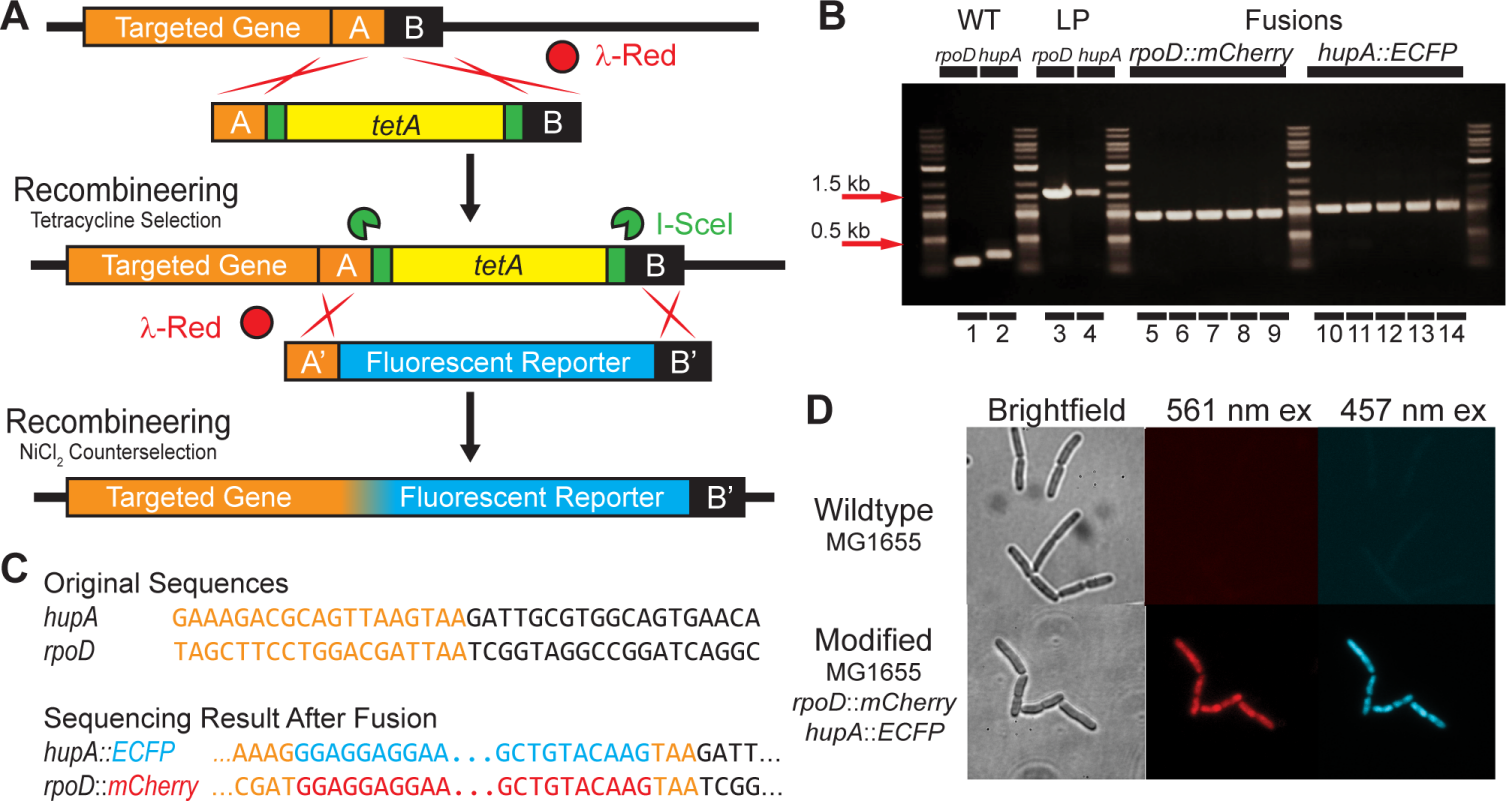
*In situ* gene fusion. (A) Strategy for gene fusion *in situ*. The *tetA* landing pad is integrated directly between the stop codon and the next base pair by homologous recombination between regions A and B, where the last 3 bp of region A is the stop codon of the targeted gene. The landing pad is then replaced by recombineering and counterselection using NiCl_2_, removing the stop codon and fusing the two coding sequences together. Homologous recombination between regions A and A’ (identical to A without TAA STOP) and B and B’ (identical to B with new TAA STOP) results in translational fusion. (B) Agarose gel electrophoresis image of colony PCR products verifying fusions to *rpoD* and *hupA*, columns 1–2: wildtype; columns 3–4: *tetA* Landing Pad (LP) integrants; columns 5–9: *rpoD*::*mCherry* fusion; columns 10–14: *hupA*::*ECFP* fusion. (C) Sequencing results for fusions of *rpoD*::*mCherry* and *hupA*::*ECFP*. (D) 400X images of wildtype MG1655 (top) and MG1655 *rpoD*::*mCherry hupA*::*ECFP* (bottom) with brightfield (left column), 561 nm laser excitation for mCherry (middle column) and 457 nm laser excitation for ECFP (right column).

To demonstrate *in situ* translational fusion of native chromosomal genes of interest to genes encoding a fluorescent reporter, we fused *mCherry* to *rpoD* (the housekeeping sigma factor σ^70^) and ECFP to *hupA* (the α subunit of the heterodimeric nucleoid associated protein HU). The strategy and results are outlined in Fig 4.

We first amplified by PCR the *tetA* landing pad from the template plasmid pTKLP-*tetA*. Landing pad amplification was primed from the standardized LP1 and LP2 priming sites. The primers were additionally designed to produce landing pad fragment flanked by the last 50 bp of the coding sequence of the targeted gene and the adjacent 50 bp of chromosomal sequence immediately downstream of the stop codon of each gene. The landing pad was then integrated and verified as described above (see *Amplification, preparation, and integration of Landing Pad*). This procedure therefore generated strains in which the *tetA* landing pad was successfully integrated exactly between the TAA stop codon of the targeted gene and the base pair immediately adjacent to TAA.

We next amplified the coding sequences of the fluorescent reporters mCherry from plasmid pRSET-B mCherry [39] and the cyan fluorescent reporter ECFP from plasmid pLAU53 [40]. The primers used were designed to amplify a linear fragment including the coding sequence of the fluorescent reporter flanked at the C terminal end by a TAA stop codon and the 50 bp of sequence immediately downstream of TAA for the targeted gene. At the N terminal end, the primer includes homology to the last 50 bp of the targeted gene *excluding the TAA stop codon*, and an additional 15 bp encoding five glycine residues as a flexible linker between the targeted gene and the fluorescent reporter gene. To complete the fusion, this PCR product was transformed into *tetA* landing pad integrants carrying pTKRED as described above (see *Preparing competent cells and transformation for recombineering*). After electroporation and recovery for four hours at 30°C, the entire culture was then added to 10 ml of RDM + 0.5% v/v glycerol with 6 mM NiCl_2_ and incubated at 37°C in a shaking water bath until saturation (usually ~48 hours). Finally, a sample of the saturated culture was diluted 10^5^ fold and plated on LB agar. Successful integrants were verified by colony PCR and sequencing.

### Scarless deletion by intrachromosomal recombination

**Fig 5:**
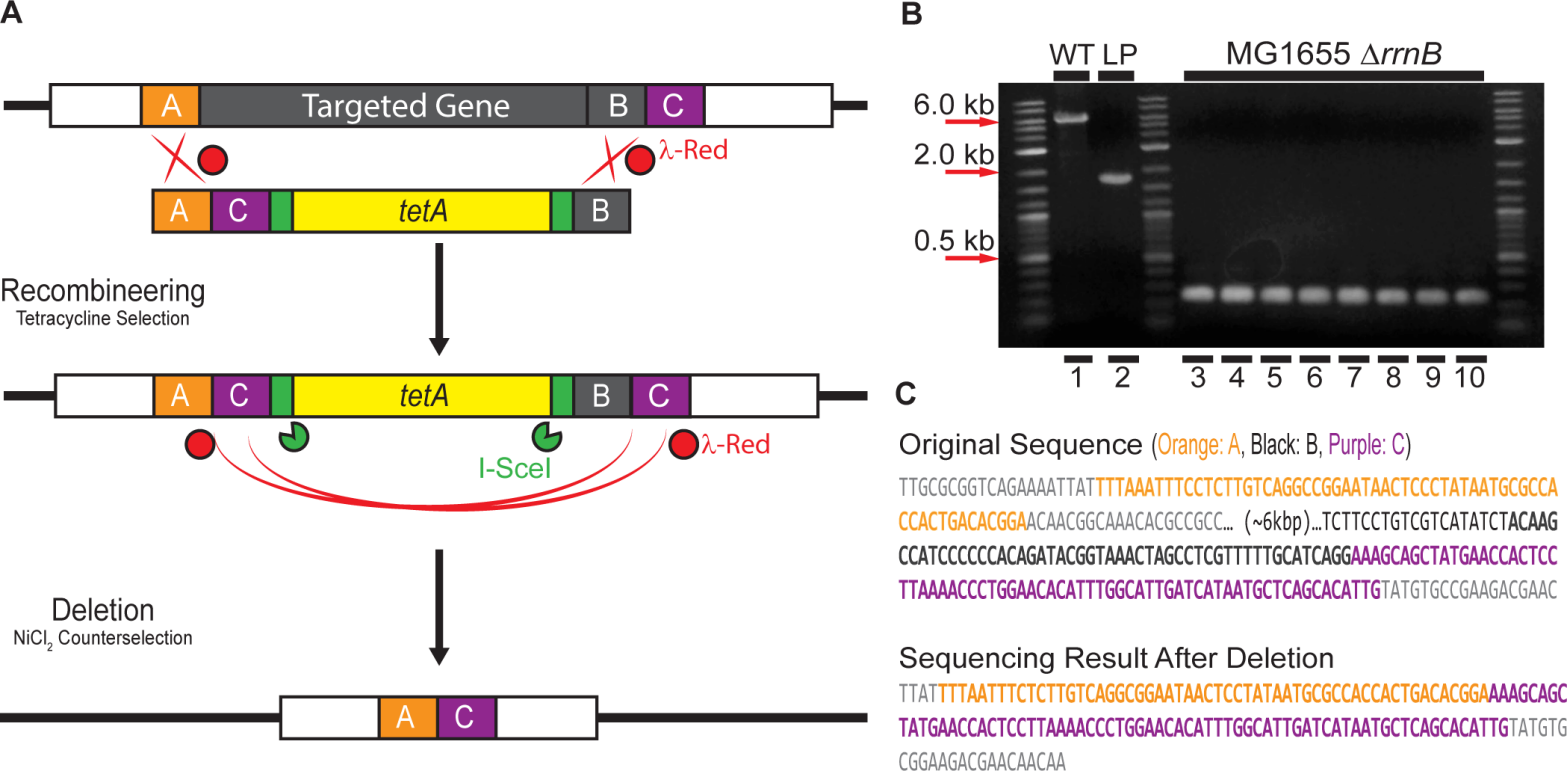
Scarless deletion by intrachromosomal homologous recombination. (A) Strategy. The target gene is replaced by recombineering using a landing pad amplified using primers containing homology regions A, B, and C. The landing pad is then eliminated by *in vivo* I-SceI digestion and λ-Red mediated homologous recombination between regions C, followed by counterselection against *tetA* landing pad retention with 6 mM NiCl_2_. (B) Verification of the deletion by colony PCR using primers flanking the *rrnB* operon. Lane 1: wildtype *rrnB* from MG1655 (WT; ~6 kbp); Lane 2: MG1655 *rrnB*::*tetA* landing pad integrant (LP; ~1.6 kbp). Lane 3–10: 8 randomly picked colonies after deletion (270bp). (C) Sequence of the operon *rrnB* and the sequencing result after deletion. Targeted homology regions are indicated by same color scheme as in (A).

The procedure for scarless deletion by intrachromosomal homologous recombination is based upon simplifications of the method of Yu et al [30]. The primers used to amplify the *tetA* landing pad for scarless intrachromosomal recombination were designed as shown in Fig 5A. Region A (50bp) and region C (75bp) consist of homology to two genes upstream and downstream of the target gene. In this case, region C is 75 bp of sequence immediately downstream of the 3’ end of target gene, the rRNA operon *rrnB*. Region B (50 bp) is the 3’ end of the targeted gene. The deletion landing pad was amplified by PCR using these primers and template pTKLP-*tetA*. This deletion landing pad includes homology regions A, B, C, the antibiotic marker *tetA*, and recognition sites for I-SceI (Fig 5A).

The deletion landing pad was then integrated into chromosome of the wildtype strain MG1655 as described previously (see *Amplification, preparation, and integration of Landing Pad*) to replace the target gene *rrnB* facilitated by λ-Red enzymes expressed from pTKRED. Colonies with deletion landing pad were selected for on LB plates containing 100 μg/ml spectinomycin and 10 μg/ml tetracycline. Colony PCR and sequencing was used to verify the presence of the deletion landing pad in the place of *rrnB*.

The scarless deletion of the landing pad was performed by growing the resulting landing pad strain with pTKRED in a 20 mm glass test tube containing 5 ml RDM + 0.5% v/v glycerol media with 100 μg/ml spectinomycin, 2 mM IPTG, and 0.4% w/v L-arabinose in a 30°C shaking water bath until saturation. 20μl of this saturated culture was then used to inoculate another 20 mm glass test tube containing 5 ml RDM + 0.5% v/v glycerol with 6 mM NiCl_2_ to eliminate cells retaining the landing pad before plating them on LB plate. Colonies were then screened on LB plates with and without 10 μg/ml tetracycline to verify the absence of the landing pad. Final verification of the deletion was performed by colony PCR and sequencing.

### Scarless deletion with oligonucleotides

Scarless deletion can also be accomplished using two sets of small oligos rather than the single set of large primers used for scarless deletion by intrachromosomal recombination. Here, the landing pad was amplified using primers for the standardized LP1 and LP2 priming sites and including 30 bp of homology to chromosomal sequence adjacent to the desired deletion. This landing pad was then integrated into the chromosome as previously described (see *Amplification, preparation, and integration of Landing Pad*), and the integration was verified by colony PCR and sequencing.

**Fig 6:**
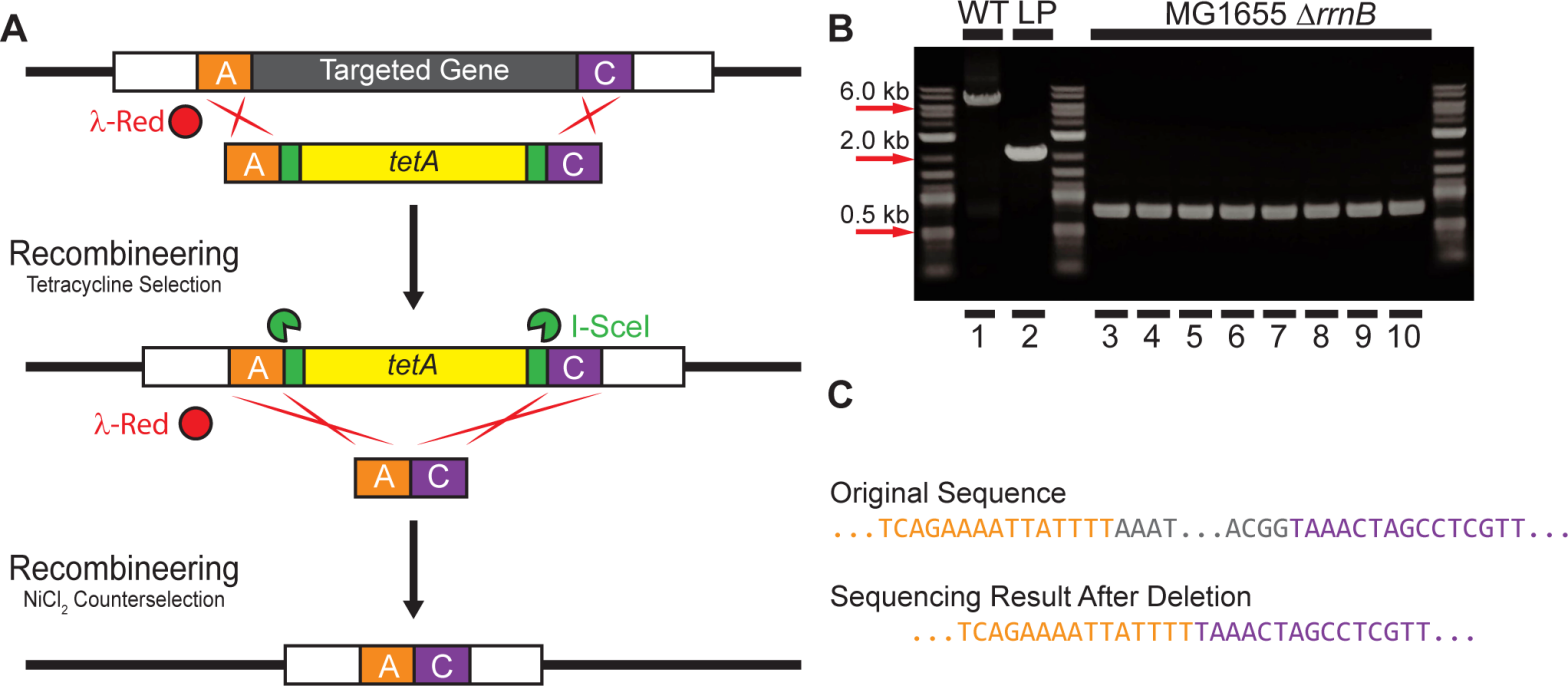
Scarless deletion by recombineering with oligonucleotides. (A) Strategy. The target gene is replaced by recombineering with the *tetA* landing pad. The landing pad is then replaced by recombineering with short, synthesized oligonucleotides and counterselection with 6 mM NiCl_2_. (B) Agarose gel electrophoresis of colony PCR products verifying deletion of *rrnB*. Lane 1: Wildtype *rrnB* (WT); Lane 2: *tetA* landing pad integrant; Lanes 3–10 8 randomly sampled colonies after deletion. (C) Sequencing result after deletion compared to the original sequence. Targeted homology regions are indicated by the same color scheme as in (A).

Scarless deletion of the landing pad was then performed by transformation of the cells with small oligonucleotides consisting solely of the two 30 bp homology sequences included in the landing pad primers (Fig 6A). The landing pad strains with pTKRED were made competent and transformed with equimolar amounts of oligos as described above (see *Preparing competent cells and transformation for recombineering*). After electroporation, 1 ml of SOB with 2 mM IPTG and 0.4% w/v L-arabinose was added, and the culture was allowed to recover for four hours. The entire culture was then added to 10 ml of RDM + 0.5% v/v glycerol with 6 mM NiCl_2_ and incubated at 37°C in a shaking water bath until saturation (usually ~48 hours). Finally, a sample of the saturated culture was diluted 10^5^ fold and plated on LB agar. Successful integrants were verified by screening for appropriate antibiotic resistances, followed by colony PCR and sequencing.

## Results

### NiCl_2_ counterselection against *tetA*

To increase the efficiency of isolation of successfully modified bacteria, unsuccessfully modified bacteria which retain *tetA* after the attempted excision of the landing pad by I-SceI can be selected against using NiCl_2_ [31]. We have verified and quantified the efficacy of *tetA* counterselection using NiCl_2_ in *E*. *coli* MG1655 within the context of the Landing Pad system by growing wildtype and *rrnB*::*tetA* landing pad integrants (see Methods, *Scarless Deletion*) in a spectrum of NiCl_2_ concentrations from 0–10 mM (Fig 2). The large error bars on the data points at high nickel chloride concentrations are a consequence of fitting an exponential growth model to noisy growth curves resulting from NiCl_2_ severely impacting exponential growth. However, our results indicate that wildtype and landing pad integrants grow well at low NiCl_2_ concentrations, and are both completely arrested at high concentrations. However, wildtype MG1655 is able to grow satisfactorily in a range of 5–7 mM NiCl_2_, while the growth of *tetA*-expressing landing pad integrants is arrested at these concentrations.

Because of the higher average copy number of genes located near the origin of replication, *oriC*, the absolute level of *tetA* expression will vary as a function of the location of integration relative to *oriC* [38, 41–44]. However, we have verified for a variety of landing pad integration locations around the chromosome ([8, 38] and this report) that 6 mM NiCl_2_ is effective for *tetA* landing pad counterselection regardless of the integration location, as will be shown for integrations, deletions, and gene fusions in a variety of locations in the following sections.

### Exact and antibiotic-free integration

In a previous version of the Landing Pad method, two 25 bp “landing pad regions” (LP1 and LP2) were used as targets for homologous recombination [8]. However, in some instances, the introduction of these extra 25 bp regions may be undesirable, such as when fusing two genes together, or integrating a new gene into an existing operon. In such cases, it is required that the integration be exact; that is, no additional sequence without direct and desired coding function can be included in the integration fragment.

Exact integration of very large constructs can be accomplished by making minor modifications to the donor plasmid pTKDP to replace the LP regions with regions of sequence homology to the desired chromosomal integration locus [9]. To demonstrate this, we generated linear DNA fragment encoding the entire *lac* operon by PCR using primers designed so that the fragment is flanked on either end by I-SceI sites and 50 bp homology to one of three loci in the *E*. *coli* chromosome: near the chromosomal origin of replication (*atpI* locus), near the terminus of replication (*nth* locus), or halfway between the origin and terminus on the left hand replichore (*ygcE* locus) [8]. These fragments were ligated into pTKDP plasmid backbone also previously digested with I-SceI and dephosphorylated. With these two simple steps, we generated three donor plasmids containing a large integration fragment (the ~6.5 kbp *lac* operon) without any antibiotic marker and flanked by locus-specific homology regions.

These plasmids were transformed into the corresponding strains where the *lac* operon had been deleted (MG1655 Δ*lac* [8]) and where the *tetA* landing pad had been integrated at each of the three loci. The integration of the *lac* operon was performed as described in Methods, and the number of correct integrants both before and after NiCl_2_ + sucrose counterselection was quantified as the number of blue/white when grown on 5-bromo-4-chloro-3-indolyl-β-D-galactopyranoside (X-gal). Using the *tetA* landing pad, the integration is extremely efficient at the *atpI* and *nth* loci, yielding ~95–97% correct integrants even without any additional selection or counterselection. Integration at the *ygcE* locus without counterselection was less efficient, yielding ~26% successful integrants; this efficiency was increased to ~76% when counterselection with 6 mM NiCl_2_ and 5% w/v sucrose was applied. PCR amplification across the integration locus and sequencing of failed *ygcE* integrants showed that the landing pad had been excised and the break repaired by intrachromosomal nonhomologous recombination. It is unclear what the differences are between the *ygcE* locus and the *atpI* and *nth* loci that render the *ygcE* locus, but not the *atpI* or *nth* loci, amenable to such repair. For each strain, representative blue colonies for each locus were verified by colony PCR across the integration, and sequencing of these PCR products verified that the junctions between the integrated fragment and the adjacent chromosomal sequence were correct and exact.

We repeated this same procedure using MG1655 Δ*lac* Δ*galK* strains where a *galK* landing pad was integrated at each of the three chromosomal loci, and we quantified the efficiency of integration as outlined above. The number of correct integrants before counterselection was substantially lower (~6% -16%) using the *galK* instead of the *tetA* landing pad for unknown reasons. However, after counterselection with 5% w/v sucrose and 0.2% w/v DOG, the efficiency of integration at each locus using the *galK* landing pad was comparable to that obtained with the *tetA* landing pad. Again, representative blue colonies for each strain were verified by colony PCR (Fig 3) and sequencing. Therefore, using the *galK* landing pad, we have exactly integrated a ~6.5 kbp fragment into specific chromosomal loci without the introduction of any extraneous sequence and without the integration of any antibiotic markers at any step of the procedure.

### In situ gene fusion

Using recombineering and counterselection against the *tetA* landing pad, we used the Landing Pad system to demonstrate the *in situ* translational fusion of the native chromosomal genes encoding the housekeeping sigma factor σ^70^, *rpoD*, and the α subunit of the histone-like nucleoid associated protein HU, *hupA*, to fluorescent reporter genes without any additional disruption of surrounding sequence. The strategy is outlined in Fig 4A. Using recombineering and positive selection for the *tetA* landing pad with tetracycline, we first integrated the landing pad between the stop codon for the targeted gene and the next immediate base. Then, by counterselecting against landing pad retention with NiCl_2_, we replaced the landing pad with the fluorescent reporter gene, eliminating the target gene’s stop codon in the process and translationally fusing the two genes together.

Because both the coding sequences of mCherry and ECFP are short (711 bp and 720 bp respectively), replacement of the landing pad can be accomplished by direct transformation of the cells with linear DNA amplified by PCR as outlined in Methods—*in situ gene fusion*. Following counterselection in RDM medium with 0.5% v/v glycerol and 6 mM NiCl_2_, the culture was diluted 10^5^ fold and plated on LB agar. For each of the gene fusions, we picked five colonies from these plates and performed colony PCR and sequencing to verify the integrations (Fig 4B and 4C); all colonies tested had successfully integrated the fluorescent reporter gene. Finally, to demonstrate repeatability, we again performed the *hupA*::*eCFP* fusion in the previously generated MG1655 *rpoD*::*mCherry* fusion strain. The success rate for verified colonies was again 100%, and example images of both wildtype MG1655 and MG1655 *rpoD*::*mCherry hupA*::*eCFP* in the brightfield and fluorescent channels are shown in Fig 4D.

### Scarless deletion by intrachromosomal recombination

We also demonstrated the usage of Landing pad system in scarless deleting of genes. As an example, the rRNA operon *rrnB* was selected as a target gene of deletion. The deletion landing pad was first integrated into chromosome to replace *rrnB*, then the deletion of this landing pad was performed in the recombination growth with IPTG and L-arabinose (see Methods, Scarless deletion by intrachromosomal recombination)

After the NiCl_2_ selection step, we selected several colonies for screening; none grew on plates containing 10 μg/ml tetracycline. Eight colonies were picked randomly to verify the result of the deletion by colony PCR and sequencing (Fig 5B and 5C). Agarose gel electrophoresis of the colony PCR products showed that the original operon *rrnB* (size of about 6kbp) (Lane 1) was replaced by the deletion landing pad (size of 1.6 kbp) (Lane 2) after landing pad integration. After deletion and counterselection, all of eight representative colonies had the band of size 270bp demonstrating excision of the *tetA* landing pad (Fig 5B). Sequencing results of the PCR product (270bp) confirmed the desired scarless deletion of *rrnB* (Fig 5C). We have subsequently used this procedure to delete each of four other ribosomal operons (data not shown), with the same level of success.

### Scarless deletion with oligonucleotides

While scarless deletion by intrachromosomal recombination is effective and efficient, it requires the synthesis of two large, expensive primers (75 bp and 150 bp) including the homology regions A, B, and C for the targeted locus. We have demonstrated another scarless deletion method requiring lower cost by using small oligonucleotides. These oligos contain the same two homology regions used to integrate the landing pad primer synthesized directly adjacent to one another. Therefore, when these oligos are transformed into the cells and replace the landing pad by homologous recombination enhanced by I-SceI- induced double strand breaks, the entire intervening sequence is eliminated (Fig 6A). The efficiency of integration was tested with the usages of single stranded oligos targeting either the leading or lagging strand, or annealed double stranded oligos.

We prepared the landing pad integrants for recombineering and transformed them with equimolar amounts of single stranded and double stranded oligos as described in Methods—*Scarless deletion with oligonucleotides*. After transformation and recovery for four hours, the culture was transferred to 10 ml RDM + 0.5% v/v glycerol with a final concentration of 6 mM NiCl_2_ and allowed to grow to saturation for *tetA* counterselection. A sample of this culture was diluted 10^5^ fold and plated on LB agar, and 20 representative colonies for deletions with each type of oligo were verified by colony PCR and sequencing (Fig 6B and 6C). Colony PCR for all clones demonstrated that *rrnB* and the landing pad was deleted with 100% efficiency; 10 representative samples from Okazaki fragment-like lagging strand targeting single stranded oligos are shown in Fig 6B. Sequencing, however, showed that double stranded and Okazaki-like single stranded oligos deleted the operon without any error in 60% of samples, while leading stranded-targeting single stranded oligos were without error in only 2 out of 20 samples. These results are in accord with previous studies showing that recombineering is much more efficient using double or single stranded oligos that incorporate into the lagging strand during replication [45].

## Discussion

We have shown here how, using NiCl_2_ or DOG for counterselection against landing pad retention, the Landing Pad system [8, 9] can be extended to generate a wide variety of genome modifications that would previously require the application of a wide variety of editing systems. The procedures described here take ~1–2 weeks from start to verified end product. Using this single system, we have explicitly demonstrated the translational fusion of native chromosomal genes to fluorescent reporters without additional perturbation, scarless gene deletion, and the exact, markerless integration of very large constructs into any locus in the *E*. *coli* genome. Using a *galK* landing pad, we have demonstrated the integration of the very large (~6.5 kbp) *lac* operon into specific loci without the integration of antibiotic markers into the genome at any step. As far as we are aware, such a feat was previously impossible with any other existing technologies. Using judiciously designed primers, oligonucleotides, and integration locations, it is also possible to use the Landing Pad system to generate single base pair substitutions and indels into specific genes *in situ*. Furthermore, we describe upgrades to the Landing Pad system: the landing pad template pTKLP and donor plasmids pTKDP [previously referred to as pTKS/CS and pTKIP, respectively [8]] have been optimized to include modifications that eliminate the need for screening against transformation or retention of these plasmids.

We have demonstrated and quantified the efficacy of nickel chloride (NiCl_2_) as a reagent for the effective negative selection against *tetA* expression within the context of the Landing Pad system [8, 31]. By employing such negative selection against the landing pad, the efficiency of selection of exact modifications without additional antibiotic markers can be significantly increased. The *tetA* gene in the landing pad is ideal for this purpose: its successful integration into the chromosome can be positively selected for by growth in medium containing tetracycline, while its retention after replacement can be negatively selected against using fusaric acid or nickel chloride [31, 34, 46, 47]. Previous studies have shown that counterselection against *tetA* using fusaric acid can be effective if simultaneously combined with additional negative selection against the marker *sacB* when grown in the presence of sucrose [34]. However, both fusaric acid and DOG are significantly more expensive than nickel chloride, and the necessity to combine fusaric acid counterselection with *sacB* for adequate selective pressure eliminates other possible simultaneous applications of *sacB*.

To effect the exact integration of small fragments into the chromosome without the inclusion of any additional unwanted sequence (e.g. antibiotic selection markers), previously existing methods have employed the gene encoding galactokinase, *galK* [1]. Integration of *galK* into a *galK* ^-^ host can be selected for by the ability to metabolize the sugar galactose. The expression of *galK* can also be selected against by supplying the cells with the galactose analogue 2-deoxygalactose (DOG), which galactokinase phosphorylates into nonmetabolizable and ultimately lethal products. Positive and negative selection of *galK* using galactose and DOG is effective, and, since it does not confer resistance to antibiotics, provides advantages when working with pathogenic strains or species. We have therefore created a version of the landing pad employing *galK* rather than *tetA* for such applications. However, use of *galK* also has disadvantages. One must be sure that the intended recipient strain is incapable of galactose metabolism such that positive selection on galactose can be successful. Furthermore, galactose must be the only carbon source available for positive selection to be effective, and hence growth and selection must be performed in minimal medium [1]. The preparation of, and extremely slow bacterial growth on, minimal medium plates makes this approach tedious and time consuming, while positive and negative selection of *tetA* can be performed rapidly and at low cost in liquid rich medium containing NiCl_2_.

We have demonstrated the scarless deletions of large regions of the chromosome using two methods: recombineering of a complex landing pad to replace the targeted gene, followed by its *in vivo* digestion by I-SceI and λ-Red mediated repair by intrachromosomal recombination; and recombineering of a simpler landing pad to replace the targeted gene, followed by excision of the landing pad by I-SceI and replacement by recombineering with small oligonucleotides. Both methods are equally efficient and effective, and the choice of method will be dictated by where it is desired to allocate time and resources. Deletion by intrachromosomal recombination has the advantage of requiring only a single recombineering step, eliminating the work required for additional recombineering, e.g. the preparation and transformation of competent bacteria. However, the large primers required are relatively expensive. Alternatively, the deletion can be completed by an additional recombineering step with short oligonucleotides. Using this method and completing the deletion with a single-stranded Okazaki-like oligonucleotide, the complete set of primers and oligos was much less expensive, but requires the time and work entailed by the additional recombineering step.

The Landing Pad system compares favorably to other *E*. *coli* genome editing technologies, including recombineering, phage-based approaches, and CRISPR-Cas9 systems. Recombineering excels at the site-specific integration of short DNA fragments into the genome to accomplish integrations, deletions, and exact replacements [1–3, 5, 13, 24–30, 34, 45, 48, 49]. However, we find that correctly integrating fragments larger than 2.5–3 kbp is prone to nonspecific integration and can be prohibitively difficult [8]. Phage based systems [10–13, 15] have the opposite problem: they are extremely efficient at integrating large constructs into the genome, but without extensive engineering these integrations can only be performed at previously existing phage attachment sites in the genome. For the same reason, phage-based systems are generally not applicable for other genome modification tasks, such as generating deletions, knockouts, or mutations.

CRISPR-Cas9 based systems show great potential for genome editing [16–20, 50–52], and are based upon the same underlying principle as the Landing Pad system, and the transcription activator-like effector nucleases (TALENs) and zinc-finger nucleases (ZFNs) employed in eukaryotes [20–23]: genome modification can be performed or enhanced by endonuclease mediated site-specific cleavage of the genome. In the case of the Landing Pad system, this is accomplished by integrating unique I-SceI recognition sites into the desired genomic location along with the landing pad by PCR amplification of the landing pad using locus-specific primers. CRISPR-Cas9 systems have the advantage that they do not require such prior modification of the genome. Cleavage by Cas9 can be directed to any sequence in the genome, guided by short CRISPR RNAs (crRNAs) that are designed to be complementary to the targeted sequence. The weakness, however, is that current existing CRISPR-Cas9 genome editing systems require the design and implementation of unique constructs to express the guiding crRNA; for example, a new plasmid must be created to express the complementary crRNA for each desired targeted location to guide Cas9 cleavage to that locus. Here, the Landing Pad system has the advantage in that targeting different genetic loci only requires new sets of locus-specific primers to amplify the landing pad.

## Supporting Information

S1 Table Oligos used in this study. Oligos used for amplification and recombineering of the landing pad are designated with “LP”. Primers used for colony PCR verification are designated “ver”. Primers for amplifying the lac operon, mCherry, or ECFP with locus specific homology are designated as “lac”, “mCherry”, or “CFP” respectively. Finally, oligos used to complete the deletion of rrnB are designated “Pos” or “Neg”.
(DOCX)
