# Supplementary material for: An Integrated System for Precise Genome Modification in Escherichia coli
Source: PLoS One. 2015 Sep 2;10(9):e0136963. doi: 10.1371/journal.pone.0136963 (PMC4558010; doi:10.1371/journal.pone.0136963)
Supplement: S1 Table — Oligos used for amplification and recombineering of the landing pad are designated with “LP”. Primers used for colony PCR verification are designated “ver”. Primers for amplifying the lac operon, mCherry, or ECFP with locus specific homology are designated as “lac”, “mCherry”, or “CFP” respectively. Finally, oligos used to complete the deletion of rrnB are designated “Pos” or “Neg”. (DOCX) [file pone.0136963.s001.docx]

| **Table of Oligos** | |
| --- | --- |
| **Exact Integration of *lac*** | |
| atpI LP F | CAAAAAGCGGTCAAATTATACGGTGCGCCCCCGTGATTTCAAACAATAAGTACGGCCCCAAGGTCCAAACGGTGA |
| atpI LP R | ATAACGTGGCTTTTTTTGGTAAGCAGAAAATAAGTCATTAGTGAAAATATTTGGCTTCAGGGATGAGGCGCCATC |
| atpI-lac F | AGTGCCACCTTAGGGATAACAGGGTAATCAAAAAGCGGTCAAATTATACGGTGCGCCCCCGTGATTTCAAACAATAAGATCGAATGGCGCAAAACCTTTCGCG |
| atpI-lac R | AGCATCCTGCATTACCCTGTTATCCCTAATAACGTGGCTTTTTTTGGTAAGCAGAAAATAAGTCATTAGTGAAAATATAGGCATGATGCGACGCTTGTTCCTG |
| nth LP F | CTGCTTTCCGCTCAGGCGACCGATGTCAGTGTTAATAAGGCGACGGCGAATACGGCCCCAAGGTCCAAACGGTGA |
| nth LP R | CGGAAAATGTGCGTGTCGACAGCAATAGTCGGCCAGCCGAATGCAGTGTTTTGGCTTCAGGGATGAGGCGCCATC |
| nth-lac F | AGTGCCACCTTAGGGATAACAGGGTAATCTGCTTTCCGCTCAGGCGACCGATGTCAGTGTTAATAAGGCGACGGCGAAATCGAATGGCGCAAAACCTTTCGCG |
| nth-lac R | AGCATCCTGCATTACCCTGTTATCCCTACGGAAAATGTGCGTGTCGACAGCAATAGTCGGCCAGCCGAATGCAGTGTTAGGCATGATGCGACGCTTGTTCCTG |
| ygcE LP F | TCAACAGCGTATAGAGGCGGTTATGTAAAACCACTCATTAGCCTCAAAACTACGGCCCCAAGGTCCAAACGGTGA |
| ygcE LP R | GATACAAAAAATTAAATTTAATCAAAGTGTTATTTGTATGATTCTTAAATTTGGCTTCAGGGATGAGGCGCCATC |
| ygcE-lac F | AGTGCCACCTTAGGGATAACAGGGTAATTCAACAGCGTATAGAGGCGGTTATGTAAAACCACTCATTAGCCTCAAAACATCGAATGGCGCAAAACCTTTCGCG |
| ygcE-lac R | AGCATCCTGCATTACCCTGTTATCCCTAGATACAAAAAATTAAATTTAATCAAAGTGTTATTTGTATGATTCTTAAATAGGCATGATGCGACGCTTGTTCCTG |
| atpI ver F | CAGTAACTGAACGAGCAGAAG |
| atpI ver R | CTTCGTCAGGTGCAACATGAGC |
| nth ver F | ACCACCGAGCTTAATTTCAGTTCGC |
| nth ver R | CCTGTTCGACGTTTTTCCCCGGCGC |
| ygcE ver F | CGCTGATGCAGGGAACATAATAAAAAC |
| ygcE ver R | CCCCGCGCCAGCGGGGATAAACCA |

| **In Situ Gene Fusion:** | |
| --- | --- |
| For *rpoD* Gene | |
| rpoD mCherry F | GAGCCGTTCTGAAGTGCTGCGTAGCTTCCTGGACGATGGAGGAGGAGGAGGAATGGTGAGCAAGGGCGAGGAGGATA |
| rpoD mCherry R | TAGTGCCGGGTGCGGCGTAACGCCTGATCCGGCCTACCGATTACTTGTACAGCTCGTCCATGCCG |
| rpoD LP F | GAGCCGTTCTGAAGTGCTGCGTAGCTTCCTGGACGATTAATACGGCCCCAAGGTCCAAACGGTGA |
| rpoD LP R | TAGTGCCGGGTGCGGCGTAACGCCTGATCCGGCCTACCGATTGGCTTCAGGGATGAGGCGCCATC |
| rpoD ver F | ATCCGTCAGATCGAAGCGAAGGCG |
| rpoD ver R | GTTGAAGCGTATCGCGAGCTGGAC |

| For *hupA* Gene | |
| --- | --- |
| hupA CFP F | AGTGCCACCTTAGGGATAACAGGGTAATCTAACGTACCGGCATTTGTTTCTGGCAAGGCACTGAAAGACGCAGTTAAGGGAGGAGGAGGAGGAATGGTGAGCAAGGGCGAGGAGCTGT |
| hupA CFP R | AGCATCCTGCATTACCCTGTTATCCCTAAGGGGTGAAACCACCCCTTCGTTAAAACTGTTCACTGCCACGCAATCTTACTTGTACAGCTCGTCCATGCCGAGA |
| hupA LP F | CTAACGTACCGGCATTTGTTTCTGGCAAGGCACTGAAAGACGCAGTTAAGTAATACGGCCCCAAGGTCCAAACGGTGA |
| hupA LP R | AGGGGTGAAACCACCCCTTCGTTAAAACTGTTCACTGCCACGCAATCTTATTGGCTTCAGGGATGAGGCGCCATC |
| hupA ver F | GTAATTGCAGAGAAAGCAGAACTG |
| hupA ver R | TGAAACCACCCCTTCGTTAAAACT |

| **Scarless Deletion by Intrachromosomal Recombination** | |
| --- | --- |
| For *rrnB* Operon | |
| rrnB LP AC F | AGAAAATTATTTTAAATTTCCTCTTGTCAGGCCGGAATAACTCCCTATAATTGGCATTGATCATAATGCTCAGCACATTGTATGTGCCGAAGACGAACAACAATTACTCAATGCCTGGCAGTATGTACGGCCCCAAGGTCCAAACGGTGA |
| rrnB LP B R | GGGTTTTAAGGAGTGGTTCATAGCTGCTTTCCTGATGCAAAAACGAGGCTTTGGCTTCAGGGATGAGGCGCCATC |
| rrnB ver F | GTTTTAGGCTGATTTGGTTGAATGT |
| rrnB ver R | GAATAAGAACGGGTTGTCCTTCTGC |

| **Scarless Deletion with Oligos** | |
| --- | --- |
| For *rrnB* Operon | |
| rrnB Del Pos | TTGAATGTTGCGCGGTCAGAAAATTATTTTTAAACTAGCCTCGTTTTTGCATCAGGAAAG |
| rrnB Del Neg | CTTTCCTGATGCAAAAACGAGGCTAGTTTAAAAATAATTTTCTGACCGCGCAACATTCAA |
| rrnB LP F | TTGAATGTTGCGCGGTCAGAAAATTATTTTTACGGCCCCAAGGTCCAAACG |
| rrnB LP R | CTTTCCTGATGCAAAAACGAGGCTAGTTTATTGGCTTCAGGGATGAGGCGC |
| rrnB ver F | AAGAATGAAAGAGCCGCCAGATAC |
| rrnB ver R | GCGCAAACTCGCTGTAATTCTACG |

**S1 Table: Oligos used in this study.** Oligos used for amplification and recombineering of the landing pad are designated with “LP”. Primers used for colony PCR verification are designated “ver”. Primers for amplifying the lac operon, mCherry, or ECFP with locus specific homology are designated as “lac”, “mCherry”, or “CFP” respectively. Finally, oligos used to complete the deletion of rrnB are designated “Pos” or “Neg”.
